# Correlation analysis between plasma circulating tumour deoxyribonucleic acid variations and survival in patients with hepatocellular carcinoma

**DOI:** 10.3389/fonc.2025.1653475

**Published:** 2026-02-10

**Authors:** Songtao Liu, Jingjing Chen, Qiaoxin Wei, Jinhuan Wang, Fang Xiong, Jia Guo, Yang Wang, Mei Liu

**Affiliations:** Department of Oncology, Beijing You’an Hospital, Capital Medical University, Beijing, China

**Keywords:** Barcelona clinic liver cancer staging, circulating tumour deoxyribonucleic acid, correlation analysis, hepatocellular carcinoma, tumour mutation burden

## Abstract

**Objective:**

This study aimed to analyse the variation of circulating tumour DNA (ctDNA) in patients with hepatocellular carcinoma (HCC).

**Methods:**

Newly diagnosed patients with HCC admitted between March 2022 and October 2023 were prospectively enrolled. Plasma ctDNA testing was performed before treatment, and clinical information, treatment regimens and survival data were collected.

**Results:**

A total of 166 patients with HCC were enrolled, including 127 men and 39 women, with a mean age of 58.1±10.0 years. Of these, 137 patients were infected with hepatitis B virus, and 129 had underlying cirrhosis. According to the Barcelona Clinic Liver Cancer (BCLC) staging system, 74, 20, 52 and 20 patients were classified as stages A, B, C and D, respectively. Tumour mutation burden (TMB) ranged from 0 to 35.48 mutations per Mb; it increased with higher BCLC stages but decreased in stage D. The top five mutated genes were *TP53* (39.8%), *CTNNB1* (15.7%), *LRP1B* (12.0%), *ARID1A* (10.8%) and *FAT3* (9.0%). Patients with *TP53* and *LRP1B* mutations exhibited shorter survival (*p* < 0.05). Among the 54 patients who received systemic therapy, only those with *LRP1B* mutations had significantly reduced overall survival (OS) (*p* = 0.048). Cox multivariate survival analysis revealed that TMB and Child–Pugh scores were closely associated with OS. Regardless of tumour stage, *LRP1B* mutation was significantly correlated with OS in patients undergoing systemic therapy.

**Conclusion:**

Circulating tumour DNA testing demonstrated that TMB is closely associated with OS in patients with HCC. Patients with HCC with *LRP1B* mutations had significantly shorter survival when receiving systemic therapy. Circulating tumour DNA testing holds considerable value in predicting HCC prognosis and may serve as a biomarker for assessing patient outcomes.

## 1 Introduction

Hepatocellular carcinoma (HCC) is one of the most common malignant tumours in China (1). Although local treatment and systemic drug regimens are constantly being updated, the overall efficacy of therapy remains limited, with objective response rates between 14.3% and 30%. Improving the precision of HCC treatment has always been a focal point in clinical research. Circulating tumour DNA (ctDNA) refers to cell-free DNA fragments released into the bloodstream due to tumour cell apoptosis or necrosis, reflecting genomic abnormalities in tumours and often referred to as a ‘liquid biopsy’ (2, 3). It can be used for early diagnosis of HCC (4, 5), dynamically monitoring the efficacy of surgical resection and evaluating the therapeutic effects of lenvatinib and immune checkpoint inhibitors (6).

The current Barcelona Clinic Liver Cancer (BCLC) staging system has limitations in prognostic stratification for patients undergoing systemic therapy. A meta-analysis indicates that ctDNA shows great potential in HCC diagnosis. Analysis of ctDNA results from over 10,000 patients with cancer in China revealed detectable ctDNA in 73.5% of plasma samples, with a high mutation frequency of *TP53* in HCC (7–12). However, some studies have identified *TERT* gene mutations as the most prevalent (13–15). Currently, ctDNA research findings are inconsistent, and biomarkers for predicting the efficacy of systemic therapy remain unclear.

This study aims to detect ctDNA mutations in patients with HCC to evaluate potential biomarkers for prognosis, analyse their correlation with patient outcomes and treatment efficacy and provide a reference for developing personalised treatment strategies. The findings also lay the foundation for further related research.

## 2 Materials and methods

### 2.1 General data

Prospectively collected data from patients first diagnosed with HCC between March 2022 and October 2023 at Beijing You’an Hospital, Capital Medical University, were included. All patients met the diagnostic criteria of the *Guidelines for the Diagnosis and Treatment of Primary Liver Cancer* (2019 Edition). Staging was performed according to the BCLC staging system. The exclusion criteria were as follows: 1) comorbid malignancies in other systems or synchronous liver metastases, 2) previous local or systemic drug therapy for HCC, 3) comorbid immunodeficiency diseases, 4) pregnancy or lactation, 5) refusal to sign informed consent by the patient or immediate family members and 6) other conditions deemed unsuitable for inclusion by the investigators. This study was approved by the Ethics Committee of Beijing You’an Hospital, Capital Medical University (Approval No.: Jing You Ke Lun Zi [2022]035), and all patients provided informed consent signed by themselves or family members.

### 2.2 Circulating tumour deoxyribonucleic acid detection method

The tumour precision medicine 825-gene panel kit for solid tumours from Genetron Health (Beijing) Co., Ltd. was used. The sequencing depth reached 10,000×, ensuring accurate detection of low-frequency mutations. The lower limit of detection for this method was 0.1%, effectively identifying trace ctDNA mutations in plasma and providing reliable data for the analysis of genetic characteristics in HCC.

### 2.3 Treatment regimens

Based on the guideline recommendations, treatment plans were formulated through multidisciplinary consultations, considering the patient’s baseline of liver function and treatment preferences. Overall survival (OS) was defined as the period from the initiation date to the date of death or the end of follow-up, with the final follow-up date (31 December 2024) as the endpoint. The median follow-up time was 18 months. The primary endpoint was 2-year OS from treatment initiation. Patients with BCLC stage D disease are typically in the terminal stage, often accompanied by severe liver dysfunction and/or extensive tumour burden, with extremely poor prognosis and shorter survival. For these patients, the treatment goals focus on symptom relief, improving quality of life and prolonging survival rather than pursuing curative treatment. Therefore, in this study, the proportion of patients with BCLC stage D receiving systemic therapy was low, with most opting for supportive care. This treatment decision was based on comprehensive considerations of tumour stage, liver function, physical condition and patient preferences.

### 2.4 Cell line generation

HepG2 cells (CL-0103, Procell, Wuhan, China) were maintained in McCoy’s 5A medium (Gibco, Cat. 16600-082) supplemented with 10% (v/v) foetal bovine serum (FBS; Gibco, Cat. 10099-141) and 1% penicillin–streptomycin (Gibco, Cat. 15140-122) at 37 °C in a humidified atmosphere containing 5% CO_2_. Two independent single-guide RNAs (sgRNAs) targeting *LRP1B* exon 1 were designed using the CRISPick algorithm (Broad Institute). The sgRNA sequences (5′–3′) were as follows: *sgLRP1B-1*, GACATTGTGGTCGCCCGGTA; *sgLRP1B-2*, CGTGGGAGCCGACCGAGGTA. Oligonucleotides containing BbsI-compatible overhangs were phosphorylated with T4 PNK (NEB, Cat. M0201), annealed and ligated into BbsI-digested pSpCas9(BB)-2A-Puro (PX459) V2.0 (Addgene #62988). Exponentially growing HCT116 cells (2 × 10^5^ per well, 6-well plate) were transfected with 2.0 µg of PX459-sgRNA plasmid using Lipofectamine 3000 (Invitrogen, Cat. L3000-015) at a 2:1 reagent/DNA ratio according to the manufacturer’s instructions. After 24 h, cells were subcultured at low density (800 cells per 10-cm dish) and selected with 1.0 µg ml^-^¹ of puromycin (Sigma, Cat. P8833) for 72 h. Surviving single colonies were isolated using cloning cylinders (Sigma, Cat. Z37, 104-4) and expanded into 24-well plates.

### 2.5 Western blot

The antibodies were purchased from Santa Cruz. The cells were lysed by Passive Lysis Buffer (25 mM Tris-HCl, 150 mM NaCl, 1% NP40) containing a protease inhibitor cocktail (Roche). Total protein was determined by BCA assay (Thermo), and an appropriate amount of denatured protein with Laemmli loading dye was loaded onto 6% or 10% polyacrylamide gel for SDS-PAGE. Subsequently, a polyvinylidene difluoride membrane was used for transfer; tris-buffered saline–Tween 20 buffer containing 5% bovine serum albumin with 0.02% sodium azide was used in membrane blocking and antibody incubations. For Western Blot, all primary antibodies were used in a 1:1,000 dilution, and all secondary antibodies were used in a 1:5,000 dilution. Bound antibodies were visualised using a chemiluminescent substrate kit purchased from Tanon and GE.

### 2.6 Methyl thiazolyl tetrazolium assay for cell proliferation

Cells were plated on 96-well plates at a density of 1,000 cells per well in triplicate and incubated for 1–10 days. Methyl thiazolyl tetrazolium (MTT) (thiazolyl blue tetrazolium, from Sigma) was added into each well at a final concentration of 0.5 mg/ml, and the plates were incubated at 37°C for an additional 4 h. After incubation, all the medium was removed, and 100 µl of dimethyl sulfoxide was added to each well. The test-ready plate was then assayed using a Biotek Synergy H1 microplate reader at OD_490_. A growth curve was drawn according to OD_490_ values by day.

### 2.7 Follow-up outcome

Overall survival was defined as the period from the initiation date to the date of death or the end of follow-up, with the final follow-up date (31 December 2024) as the endpoint. The median follow-up time was 18 months.

### 2.8 Statistical methods

Statistical analysis was performed using SPSS 22.0 software. The *χ*² test or Fisher’s exact test was used to analyse the relationship between gene mutations and clinical prognosis. Survival curves were plotted using the Kaplan–Meier method and compared using the log-rank test. Univariate and multivariate Cox proportional hazards regression models were employed to assess risk factors for survival. In the Cox regression model, the following covariates were predefined: BCLC stage, Child–Pugh score, tumour mutation burden (TMB) and mutation status of key genes (e.g. *TP53*, *LRP1B*). When entered into the model, the mutation state of key genes was set as a binary variable. The sample size was calculated based on statistical power to meet research requirements. In the survival analysis, data from 28 patients lost to follow-up were censored, meaning their last follow-up time was used as the endpoint, and their survival time was included in the analysis without being counted as death events. A *p*-value <0.05 was considered statistically significant. Considering the multiple correction, a *p*-value of the key gene mutation state <0.05/*n* was considered statistically significant.

## 3 Results

### 3.1 Clinicopathological data

This study included a total of 166 patients, comprising 127 men and 39 women, with an age range of 32–86 years (mean age 58.1±10.0 years) and a median age of 58.0 years. Among them, 137 had hepatitis B virus (HBV) infection and 129 had underlying liver cirrhosis. Based on the BCLC staging system, 74, 20, 52 and 20 patients were classified as stage A, B, C and D, respectively (**Table 1**).

**Table 1:** Baseline characteristics of 166 HCC patients.

Variable	All patients (n=166)
male/female	127/39
age	58.1±10.0
HBV/alcohol/others	137/13/16
cirrhosis (yes/no)	129
BCLC (A/B/C/D)	74/20/52/20
AFP	40.6 (5.1, 1944.5)
Child-pugh score	6.0 (5.0, 8.0)

HBV, Hepatitis B virus; AFP, Alpha-fetoprotein.

### 3.2 Circulating tumour deoxyribonucleic acid test results

The TMB of 166 patients ranged from 0 to 35.48 mutations per Mb (muts/Mb). A clinically relevant cut-off of 10 muts/Mb was used to define high TMB, as established in recent trials (16, 17). The TMB increased with higher BCLC stages; the median TMB was 0.01 (0.00, 2.69) muts/Mb in stage A, 3.77 (0.27, 7.53) muts/Mb in stage B, 7.00 (4.30, 10.75) muts/Mb in stage C and 4.84 (1.08, 9.95) muts/Mb in stage D. There was a statistically significant difference among the groups (*p* < 0.001), although no significant difference was observed between stages B and D (*p* = 0.633).

**Figure 1:**
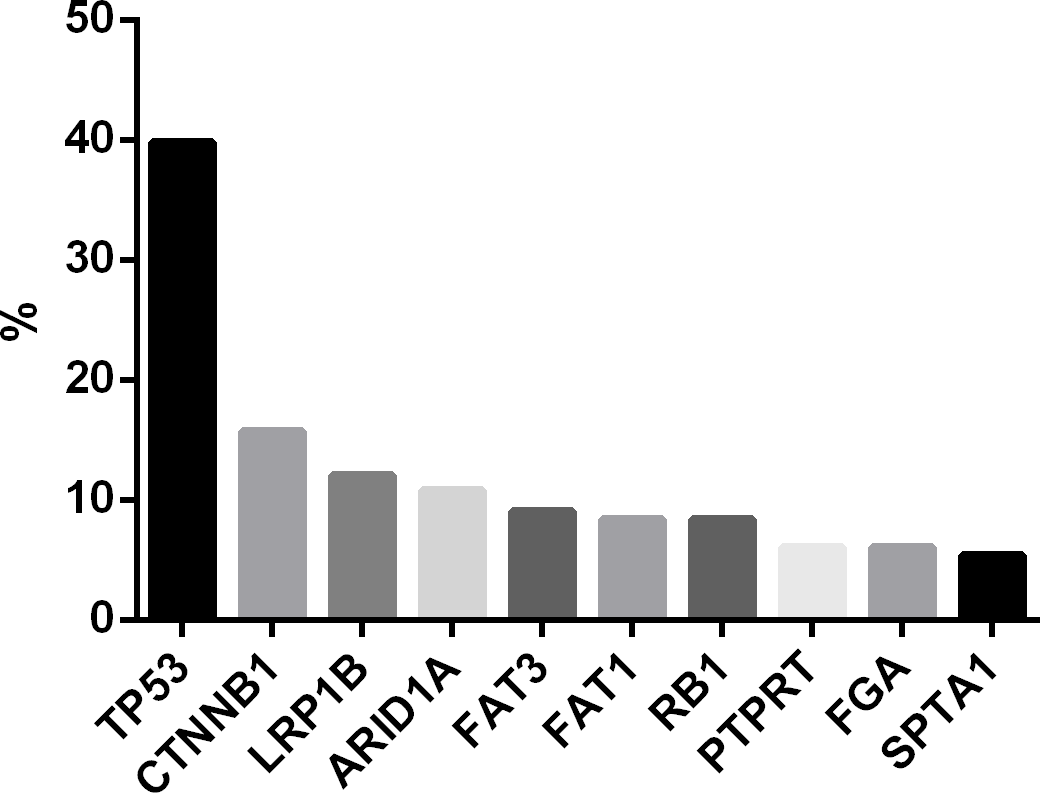
Top 10 mutated genes in HCC patients (n=166). Mutation frequencies were determined using 825-gene panel sequencing at 10,000× coverage. Values represent percentage of patients with detectable mutations in each gene.

Among the mutated genes, the top 10 most frequently mutated genes were *TP53* (39.8%), *CTNNB1* (15.7%), *LRP1B* (12.0%), *ARID1A* (10.8%), *FAT3* (9.0%), *FAT1* (8.4%), *RB1* (8.4%), *PTPRT* (6.0%), *FGA* (6.0%) and *SPTA1* (5.4%) (**Figure 1**).

### 3.3 Treatment strategies for hepatocellular carcinoma

Among the 166 patients classified by BCLC staging, the strategies were as follows: Stage A (74 patients) – 39 patients underwent surgical resection, including 4 who received systemic therapy due to postoperative recurrence and 1 who underwent combined interventional and ablation therapy for recurrence; 31 patients received combined interventional and ablation therapy, including 2 who also received systemic therapy; 1 was treated with lenvatinib, which was later switched to donafenib combined with sintilimab and a bevacizumab biosimilar due to poor efficacy; 3 refused any local treatment and were discharged. Stage B (20 patients) – 13 underwent interventional therapy, including 3 who received sequential ablation therapy and 10 who received sequential systemic therapy; among the remaining 7 patients, 4 chose systemic therapy, 2 opted for symptomatic treatment and 2 selected traditional Chinese medicine. Stage C (52 patients) – 15 received interventional therapy, including 7 who underwent sequential systemic therapy; 2 underwent surgical resection, both followed by systemic therapy; 21 received systemic therapy, and 14 opted for symptomatic treatment. Stage D (20 patients) – 2 chose systemic therapy, and the remaining 18 received supportive care.

### 3.4 Overall survival

**Figure 2:**
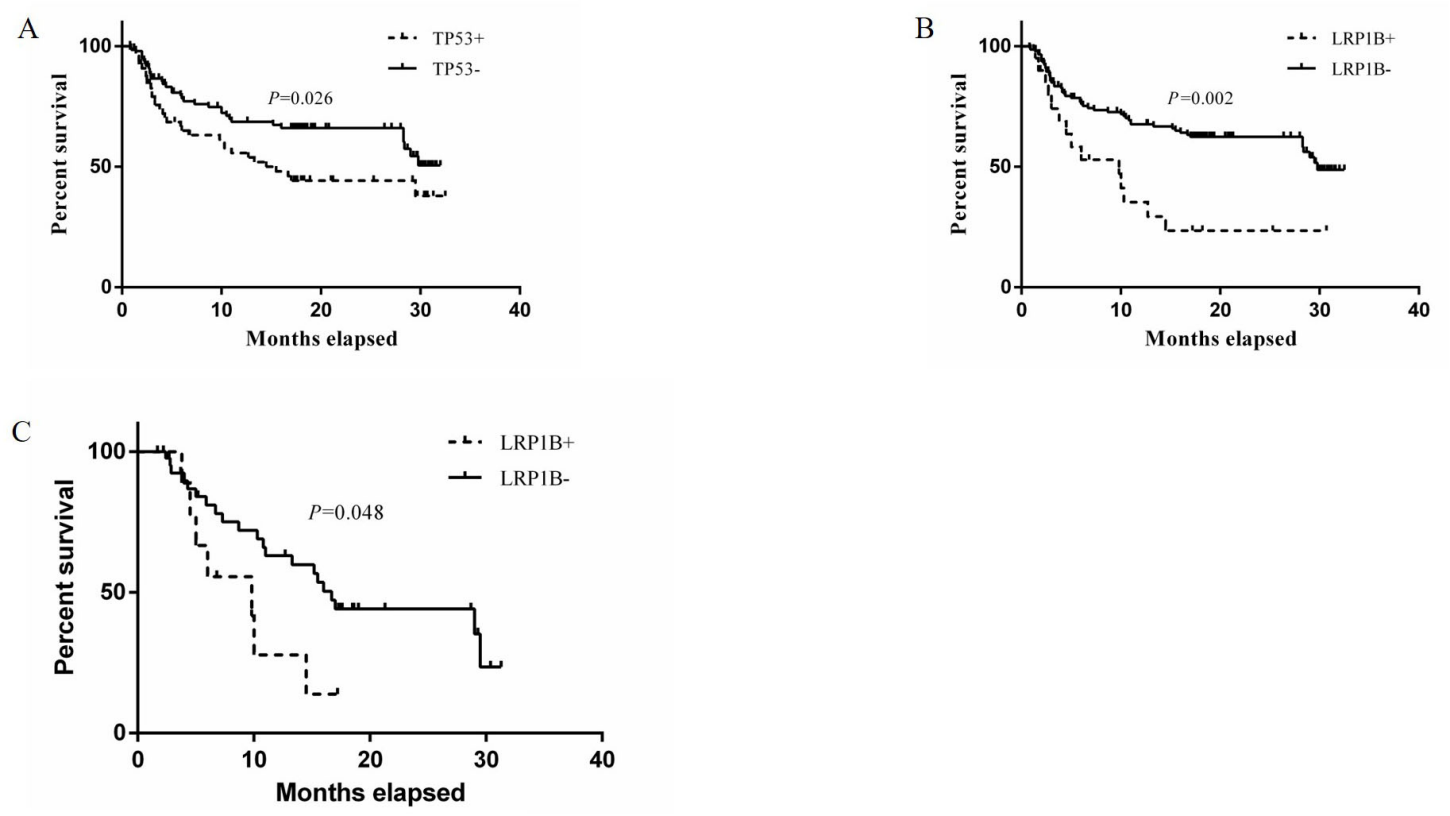
Kaplan-Meier survival curves stratified by **(A)** TP53 and **(B)** LRP1B mutation status, and **(C)** LRP1B in systemic therapy subgroup. Log-rank test P-values shown. Tick marks indicate censored patients.

As of 31 December 2024, 67 patients had died, 28 were lost to follow-up and 71 remained alive. The OS ranged from 0.8 to 32.5 months, with a median survival of 13.0 (3.0, 19.7) months. Among the top 10 mutated genes (10 binary variables), the median OS was 14.5 months (95% confidence interval [CI]: 7.7–21.3 months) in the TP53+ patients and not reached (95% CI: 28.3–n.a.) in the TP53- (P = 0.026, **Figure 2A**). The median OS was 9.8 months (95% CI: 3.1–16.5) in the LRP1B+ patients and 29.8 months (95% CI: 28.3–n.a.) in the LRP1B - (P = 0.002, Figure 2B). but no significant differences were observed for the other genes (p > 0.05/10). Among the 54 patients who received systemic therapy, 34 received targeted therapy combined with immune checkpoint inhibitors, 3 received immune checkpoint inhibitor monotherapy and the remaining 17 received targeted therapy alone. Notably, in the exploratory analysis of 54 patients receiving systemic therapy, those with LRP1B+ showed shorter median OS with 9.8 months (95% CI: 0.5–19.1) than LRP1B- with 16.7 months (95% CI: 14.3–19.1) (unadjusted p = 0.048, **Figure 2C**).

### 3.5 Factors influencing survival

The BCLC stage is closely associated with the survival of patients with HCC. In addition, Cox multivariate survival analysis revealed that TMB and Child–Pugh score are correlated with the OS of patients with HCC (**Table 2**).

**Table 2:** COX Regression Analysis of Factors Affecting Survival in 166 HCC Patients.

Variable	Overall survival				Survival of systematic drug treatment			
	*P*	Exp(B) HR (95% CI)	*P*	Exp(B) HR (95% CI)	*P*	Exp(B) HR (95%CI)	*P*	Exp(B) HR (95%CI)
TP53	0.026	1.717 (1.066-2.766)			0.809	0.911 (0.426-1.947)		
LRP1B	0.002	2.513 (1.388-4.549)			0.048	2.452 (1.008-5.963)	0.048	2.452 (1.008-5.963)
TMB	0.000	1.075 (1.045-1.106)	0.000	1.070 (1.289-1.552)	0.081	1.041 (0.995-1.089)		
Child-pugh score	0.000	1.416 (1.293-1.551)	0.000	1.415 (1.289-1.552)	0.190	1.121 (0.945-1.331)		

CI, Confidence interval.

### 3.6 *LRP1B* suppresses tumour cell proliferation and reduces lenvatinib resistance

**Figure 3:**
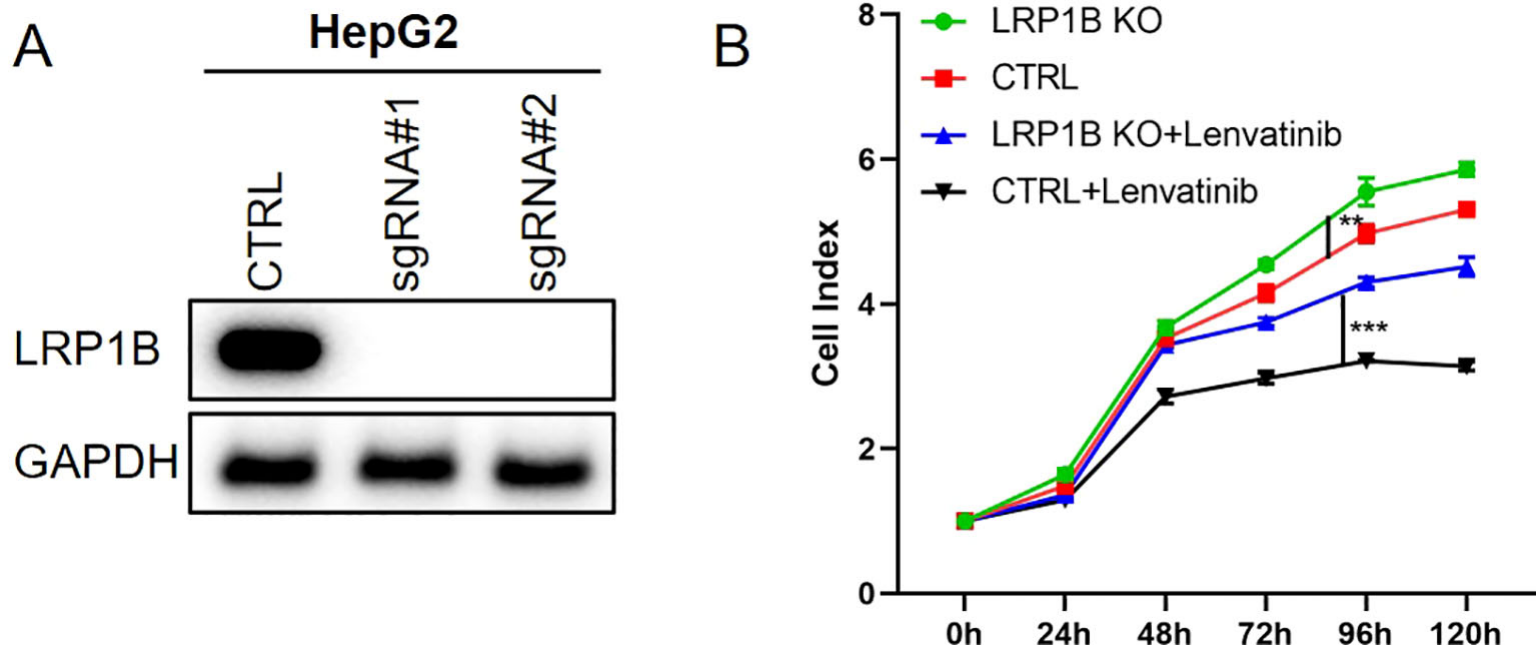
The role of LRP1B in HCC cells. **(A)** The westernblot results of LRP1B knockout. **(B)** The MTT results of HepG2 with lenvatinib (30 μM) treatment. **p<0.01, ***p<0.001.

To further validate the role of *LRP1B*, the CRISPR/Cas9 system was used to knock out *LRP1B* in the HCC cell line HepG2 (**Figure 3A**). Subsequently, MTT proliferation assays were performed using the *LRP1B*-knockout cell line. The results showed that knockout of *LRP1B* enhanced the proliferation rate of the HCC cells and increased their resistance to lenvatinib (**Figure 3B**).

## 4 Discussion

This study detected ctDNA in the plasma of patients with HCC and found that TMB gradually increased with tumour stage progression and was closely associated with patient survival. Tumour mutation burden is a valuable indicator of somatic mutation accumulation, calculated as the number of somatic mutations per million base pairs of genomic material, and is related to tumour biological behaviour and prognosis (18). Moreover, higher TMB can predict postoperative recurrence in patients with HCC (19). Due to the insidious nature of HCC and the lack of specific symptoms, although ctDNA mutation analysis can identify HCC-associated mutated genes and mutation frequencies, the accuracy and reliability of early diagnosis are inconsistent due to variations in TMB strength (20).

Whether ctDNA can reflect the characteristics of tissue samples remains controversial across studies. In one study of 17 patients, the gene mutations in adjacent non-tumour tissues were consistent with those in tumour tissue DNA. Further analysis revealed that the mutant allele frequency (MAF) of genes such as *TP53*, *CTNNB1*, *PIK3CA* and *CDKN2A* was higher in patients with tumour diameters >5 cm than in those with diameters <5 cm. In patients with multiple tumours or metastases, the MAF of *TP53*, *RET*, *FGFR3* and *APC* genes was significantly higher than in those with single tumours (21). Another study found that 71% of patients had mutations in HCC tissue DNA that were not detected in matched ctDNA. Research indicates that plasma ctDNA testing has high specificity but low sensitivity in matching HCC tissue mutations (22). Additionally, dysregulated alternative RNA splicing events, such as intron retention and exon skipping, have been implicated in HCC progression and therapeutic resistance, influencing the expression of pro- and anti-apoptotic isoforms of genes such as *MCL1* and *BCL2*, which may further complicate the molecular landscape of HCC (23). Therefore, in patients with HCC, somatic mutations – and even ctDNA-detected mutations – may differ from those in tumour tissue. The discrepancy between ctDNA and tissue may arise because not all tumour cells simultaneously release DNA into the bloodstream, making ctDNA potentially more suitable for patients with advanced HCC (24–26). However, studies have found inconsistencies in mutated genes, suggesting that consensus on ctDNA testing in patients with HCC remains elusive and requires further research to identify more patterns.

Some studies suggest that the presence or absence of ctDNA cannot predict the efficacy of atezolizumab combined with bevacizumab treatment (14). Among the mutated genes, research has shown that *TERT* mutations and alpha fetoprotein ≥400 ng/mL are independent predictors of shorter survival (14). This study found a very low mutation rate in the *TERT* gene but a high mutation rate in the *LRP1B* gene. The *LRP1B* gene is located on chromosome 2q and is widely expressed in various normal human tissues. It is also one of the most frequently mutated genes in multiple cancer types. An analysis of cancer gene mutation rates in 11,948 Chinese patients with cancer revealed that *TP53* (51.4%), *LRP1B* (13.4%), *PIK3CA* (11.6%), *KRAS* (11.1%), *EGFR* (10.6%) and *APC* (10.5%) were the most frequently mutated cancer genes (27). By contrast, the most common gene mutations in cancers in the USA are *TP53* (34.5%), followed by *PIK3CA* (13.5%) and *LRP1B* (13.1%) (28). This study found that patients with mutations in *TP53*, *CTNNB1*, *ARID1A* or *LRP1B* had poorer prognoses. However, among patients receiving systemic drug therapy, only those with *LRP1B* mutations exhibited a worse prognosis, whereas other genes showed no significant difference. The *LRP1B* gene can directly bind to the nicastrin protein, affecting its expression level and thereby regulating the PI3K/AKT pathway. Knockout of the *LRP1B* gene promotes HCC cell proliferation and migration and enhances tumour cell resistance to doxorubicin liposomes (29). However, another study showed that downregulating *LRP1B* expression significantly inhibited the proliferation, migration and invasion of HCC cells and increased their sensitivity to doxorubicin, a process mediated by the endoplasmic reticulum stress pathway (30). Thus, the role of the *LRP1B* gene in HCC requires further investigation.

In The Cancer Genome Atlas (TCGA) and International Cancer Genome Consortium datasets, *LRP1B* is one of the most frequently mutated genes in HCC cohorts. Survival curves indicate that *LRP1B* mutations are associated with poorer survival outcomes, and this correlation remains significant after adjusting for multiple confounding factors such as age, sex, tumour stage, *BRCA1*, *BRCA2* mutations and *POLE*. This suggests that *LRP1B* mutations may aid in immunotherapy selection and prognosis prediction for HCC (31). The TCGA data show that patients with *LRP1B* mutations have significantly higher TMB than those with wild-type *LRP1B*, and the *LRP1B* mutation group exhibits elevated TMB and mast cell infiltration in tumour tissue (32). In patients with HCC undergoing liver transplantation, mast cells were detected in 93% of HCC tumours and peritumoral tissues. Patients lacking intratumoral mast cells had larger tumours and higher recurrence rates, suggesting that hepatic mast cells may participate in anti-tumour immunity in HCC (33). Correlation analysis revealed that *LRP1B* mutation status is associated with increased infiltration of two types of immune cells and elevated expression of the immune checkpoint gene *HHLA2* in patients with HCC. Mutations of the *LRP1B* gene may serve as a predictor for patients with HCC with high TMB and high *HHLA2* expression (34). In both the TCGA cohort and a Chinese cohort, *LRP1B* mutations were significantly associated with higher TMB. Research findings indicate that *LRP1B* or *TP53* mutations are linked to higher TMB and poor prognostic factors in HCC (9).

In this study, we found that TMB was closely associated with the OS of patients with HCC, with higher TMB correlating with poorer prognosis. This result appears contradictory to some studies suggesting that high TMB predicts better responses to immunotherapy. However, this discrepancy may be due to the low proportion of patients receiving immunotherapy in this study. Among the 54 patients who underwent systemic drug therapy, only 3 (5.6%) received immune checkpoint inhibitor monotherapy. This indicates that most patients in this study were not treated with immunotherapy but rather with other therapeutic modalities. In this context, the association between high TMB and poor prognosis may reflect the biological significance of TMB in non-immunotherapy scenarios. In non-immunotherapy settings, high TMB may indicate greater genomic instability in tumour cells, leading to a higher likelihood of resistance mutations and, consequently, poorer treatment outcomes and prognosis. Thus, TMB may play different roles depending on the treatment context. This finding suggests that in clinical practice, TMB cannot be simplistically regarded as a single prognostic marker but must be evaluated in conjunction with specific treatment regimens. Future research should further explore the mechanisms of TMB in different treatment modalities and develop more precise biomarkers to guide clinical decision-making.

However, this study has certain limitations. First, the sample size was relatively small, with only 166 patients with HCC included. Future studies should expand the sample size to evaluate the relationship between ctDNA mutations and HCC prognosis more comprehensively. Second, this study is limited by its single-centre, single-arm design and the absence of a control group, which restricts the ability to compare the prognostic effects of mutations between patients with HCC and those without or between mutated and non-mutated patients. Future studies should include control groups to validate these findings and improve the robustness of the conclusions. Third, the majority of patients in this study (137 patients) had HBV-related HCC, with few patients attributed to other aetiologies (e.g. alcohol, other viruses). This may limit the applicability of the findings to patients with non-HBV-related HCC. Fourth, our exploratory finding that *LRP1B* mutations were associated with poorer survival in systemic therapy gains biological plausibility from recent evidence showing that *LRP1B* knockdown promotes HCC drug resistance via PI3K/AKT pathway activation (29), although validation in larger cohorts is needed to confirm clinical significance. Fifth, although advanced ctDNA detection technology was used in this study, some tissue mutations were not detected in ctDNA. Future research should further optimise ctDNA detection technology to improve its sensitivity and specificity for better clinical application. Finally, we acknowledge that the relationship between TMB and BCLC stage was assessed descriptively. A more comprehensive multivariate analysis, although valuable, was not undertaken due to our study’s sample size and primary focus on prognostic mutations, avoiding potential overfitting.

## 5 Conclusion

This study found that ctDNA detection may be a promising liquid biopsy method for predicting the prognosis of HCC. It not only serves diagnostic purposes but also plays a key role in analysing and assessing patient prognosis. However, current research findings are inconsistent, and due to the lack of standardised detection methods, there are still many limitations in its clinical application. In future research, efforts should focus on detecting a select few mutation genes that influence prognosis to improve the accuracy of diagnosis and prognostic prediction.

## Data Availability

The original contributions presented in the study are included in the article/supplementary material. Further inquiries can be directed to the corresponding author.
